# Association of helicopter transportation and improved mortality for patients with major trauma in the northern French Alps trauma system: an observational study based on the TRENAU registry

**DOI:** 10.1186/s13049-020-00730-z

**Published:** 2020-05-12

**Authors:** Francois-Xavier Ageron, Guillaume Debaty, Dominique Savary, Frederic Champly, Francois Albasini, Pascal Usseglio, Cécile Vallot, Samuel Galvagno, Pierre Bouzat, for the TRENAU Group, Francois-Xavier Ageron, Pierre Bouzat, François Albasini, Frederic Champly, Laurent Chapiteau, Etienne Haller, Christophe Hoareau, Albrice Levrat, Elisabeth Rancurel, Dominique Savary, Jean-Marc Thouret, Pascal Usseglio, Sophie Muller, Claire Vallenet, Cecile Vallot, Damien Venchiarutti

**Affiliations:** 1 Northern French Alp Emergency Network, Centre Hospitalier Annecy Genevois, 1, avenue de l’hopital - BP 90074, F-74374 Pringy, France; 2 Emergency Department, Lausanne University Hospital, CHUV, Lausanne, Switzerland; 3 Emergency Department and Mobile Intensive Care Unit, Grenoble Alpes University Hospital, Grenoble, France; 4 Emergency Department, Centre Hospitalier Annecy Genevois, Pringy, France; 5 Emergency Department, Hôpitaux du Pays du Mont-Blanc, Sallanches, France; 6 Emergency Department, Centre Hospitalier de Saint-Jean de Maurienne, Saint-Jean de Maurienne, France; 7 Emergency Department, Centre Hospitalier Metropole de Savoie, Chambery, France; 8 R Adams Cowley Shock Trauma Center, University of Maryland School of Medicine, Baltimore, USA; 9 Department of Anaesthesiology and Critical Care, Grenoble Alpes University Hospital, 38000 Grenoble, France

**Keywords:** Emergency medical services, Helicopter, Mortality, Trauma, Triage

## Abstract

**Background:**

Prompt prehospital triage and transportation are essential in an organised trauma system. The benefits of helicopter transportation on mortality in a physician-staffed pre-hospital trauma system remains unknown. The aim of the study was to assess the impact of helicopter transportation on mortality and prehospital triage.

**Methods:**

Data collection was based on trauma registry for all consecutive major trauma patients transported by helicopter or ground ambulance in the Northern French Alps Trauma system between 2009 and 2017. The primary endpoint was in-hospital death. We performed multivariate logistic regression to compare death between helicopter and ground ambulance.

**Results:**

Overall, 9458 major trauma patients were included. 37% (*n* = 3524) were transported by helicopter, and 56% (*n* = 5253) by ground ambulance. Prehospital time from the first call to the arrival at hospital was longer in the helicopter group compared to the ground ambulance group, respectively median time 95 [72–124] minutes and 85 [63–113] minutes (*P* < 0.001). Median transport time was similar between groups, 20 min [13–30] for helicopter and 21 min [14–32] for ground ambulance. Using multivariate logistic regression, helicopter was associated with reduced mortality compared to ground ambulance (adjusted OR 0.70; 95% CI, 0.53–0.92; *P* = 0.01) and with reduced undertriage (OR 0.69 95% CI, 0.60–0.80; *P* < 0.001).

**Conclusion:**

Helicopter was associated with reduced in-hospital death and undertriage by one third. It did not decrease prehospital and transport times in a system with the same crew using both helicopter or ground ambulance. The mortality and undertriage benefits observed suggest that the helicopter is the proper mode for long-distant transport to a regional trauma centre.

## Background

Injuries contribute significantly to the global burden of disease with more than 5 million deaths each year [1]. The severely injured patient needs to be promptly treated in an appropriate medical facility: “the right treatment in the right place at the right time” [2]. Several studies have shown a benefit of the direct admission of major trauma in a highly specialized facility designated as a regional trauma centre [3]. Failure to admit patients with major trauma in a dedicated trauma centre may lead to inappropriate care and consequently an increase of preventable deaths [4].

Helicopters represent a transportation modality that enables expeditious transport to trauma centres and in most developed parts of the world, helicopter exists as an integral part of an organized trauma system [5]. Nevertheless, the effectiveness of helicopter remains controversial [6, 7]. In 2015, a review including 38 studies was not able to firmly assert a mortality benefit owing to methodological weaknesses and significant heterogeneity in the available literature [8, 9]. However, when this work is carefully examined, helicopter demonstrated a consistent mortality benefit in studies that used multivariate models to control for known confounders. Several hypotheses have been stated to explain the benefits associated with helicopter [10]. Rapid transportation, crew expertise and the role of helicopter as an integrated part of trauma system represent cogent explanations. The effectiveness of helicopter in the Northern French Alps trauma system has yet to be rigorously studied. Since both ground ambulance and helicopter are staffed by emergency physicians, there exists a unique opportunity to examine the clinical effectiveness of helicopter while controlling for one of the most important confounding factors that has plagued previous studies: crew expertise. The objective of the present study is to assess the impact of helicopter transportation on in-hospital death in a system where crew expertise is not different between ground ambulance and helicopter.

## Methods

### Study setting

The Trauma system of the Northern French Alps Emergency Network (TRENAU) was implemented in 2009 as part of the Northern French Alps Emergency Network (RENAU). This system was created in 2000 with the aim of developing various programs to address emergency medical conditions including care for the traumatically injured. TRENAU has been described previously [11–13]. Briefly, all 24 hospitals with an emergency department and three EMS systems provide network coverage in a mountainous area of 18.000 km^2^ across three counties (Haute-Savoie, Savoie and Isère) (Additional file 1).

The population covered by the network is inhabited by more than 2 million with a large seasonal variation (more than 8 million tourists each year). Thirteen hospitals were designated to admit major trauma patients with one regional level I trauma centre at the University Hospital of Grenoble and one additional level I trauma centre at General Hospital of Annecy to support admission of severely injured patients in the northern region.

The EMS system in France is a two-tiered system with basic life support ambulances staffed by fire departments, and advanced life support ambulances staffed by emergency physician (Mobile Intensive Care Units) [14]. For all potentially life-threatening conditions, two ambulances are dispatched: one with basic life support and one with advanced life support. Helicopter is integrated in some mobile intensive care units when a helicopter is available and uses the same personnel and the same medical equipment as the advance life support ambulances. Thirteen mobile intensive care units are located in the Northern French Alps, one in each designated hospital and available continuously. Six of these units use helicopters and in total, seven helicopters are available during daytime hours (Additional file 1). Six helicopters have different pre-defined uses to include helicopter as well as Search and Rescue (SAR) services. One helicopter is dedicated to medical response only and located in the regional level I trauma centre in Grenoble. Helicopters are typically available during daytime hours pending weather restrictions but may also be dispatched at night with half an hour of delay due to preparation for night time operations.

### Population

The study population was comprised of all injured patients with major trauma in the Northern French Alps and recorded prospectively in the TRENAU registry. Major trauma was defined by the Vittel’s Criteria of the French Emergency Medicine Society and corresponded to the field triage decision scheme of the American College of Surgeon [15, 16]. Injured patients with at least one diagnosis with an abbreviated injury scale (AIS) equal or higher than 3 for the head, thorax, abdomen and pelvis and admitted at hospital were also included for analysis in this study if they were not already included in the prehospital setting. We excluded patients transported only by basic life support fire department ambulances, patients who arrived in the ED on their own, patients transported outside the area of the 77Northern French Alps in a hospital not included in the trauma network and patients not transported (death on scene). This study received ethical approval from the institutional review board of the University Hospital of Clermont Ferrand and the Research Committee of the London School of Hygiene and Tropical Medicine.

### Study design

The study design is a retrospective observational study based on data available in the registry of the TRENAU from 2009 and 2017 inclusive. The data collected were considered suitable for the Utstein Style Major Trauma revisited [17]. Registry collected data including all prehospital interventions and interventions provided up to hospital discharge, age, gender, mechanism of injury, circumstances related to the injury, physiological parameters, intervention times, injury severity score (ISS) and status at discharge. Data were collected initially by the in-charge physician and entered in an electronic database. Research technicians provided continuous monitoring of the completeness and the quality of the data, and collected patient outcome data at the time of hospital discharge.

### Endpoints

The primary outcome of interest for this study was in-hospital death. Secondary endpoints included assessment of field triage including the under-triage and over-triage rates, as defined by the American College of Surgeons [18]. Under-triage was defined as a severely injured patient (Injury Severity Score [ISS] ≥ 16) not admitted initially at a level I trauma centre. Over-triage was defined as injured patients with an ISS lower than 16 admitted in a specialised resuscitation room of a level I trauma centre. Additional secondary endpoints examined were response time, on-scene time, transport time and total prehospital time.

### Statistical analysis

The characteristics of the patients were examined according to the type of the transportation mode (helicopter or ground ambulance). Continuous variables were compared using either Student’s t test or the Wilcoxon rank sum test depending on the parametric or nonparametric distribution, respectively. Categorical variables were compared using Pearson’s Chi-squared test or Fisher’s exact test. As the study groups were not strictly comparable in term of severity, the Trauma and Injury Severity Score (TRISS) was used to estimate the predicted mortality rate [19]. The W score was calculated as the absolute difference of the observed and expected mortality rate for each group. As we determined an expected mortality rate by indirect standardisation, the Standard Mortality Ratio (SMR) was calculated and then compared between study groups. In addition, we performed a backward stepwise logistic regression model controlling for the type of transportation mode (helicopter or ground ambulance) and other known confounders: severity of injury with the Injury Severity Score (ISS), physiologic parameters including the Glasgow Coma Scale and systolic blood pressure, demographic characteristics (age and gender), circumstances of injury (road traffic accident, fall, penetrating injury, mountain accident), severe traumatic brain injury (Abbreviated Injury Scale ≥3), total prehospital time and prehospital oro-tracheal intubation as a proxy on intensity of prehospital care. We assessed graphically departure from linearity for continuous variable. We included polynomial terms in the model when appropriated. We removed one at a time covariates that were not significant according to the Wald test (*P* value less than 0.05), and performing a likelihood ratio test at each point. We tested all plausible interactions (between transportation mode, ISS, Glasgow coma scale, systolic blood pressure, age, circumstances of injury, prehospital oro-tracheal intubation and total prehospital time) to assess for effect modification. We included a random effect on mobile intensive care unit to control for a potential cluster effect. We choose the covariables of the multivariate model in a parsimonious approach to avoid any risk of overfitting in the model. We were careful to not include in-hospital variable to limit overadjustment bias due to variable on the causal pathway from transportation mode and death. The sample size was fixed as we used registry-based data, so a sample size was not calculated a priori; however; we estimated statistical power post hoc with the difference observed in in-hospital death. All tests were 2-tailed and a *P* value < 0.05 was considered statistically significant. All statistics were performed using Stata version 15.0 (Statacorp, College Station, TX, USA).

### Missing values

To handle missing values, we performed multiple imputation using chained equations. We reported 390 (4%) incomplete observations for the predictors used in the analysis. We imputed 20 datasets to fill in missing values for usual predictors and primary outcomes used in the multivariate model (systolic blood pressure, Glasgow coma scale, injury severity score, age, penetrating injury, death). We imputed data for prehospital times including location on mobile intensive care unit and hospital admission. We performed sensitivity analysis regarding prehospital times according to transportation type (helicopter or ground ambulance).

## Results

### Patient characteristics

**Fig. 1:**
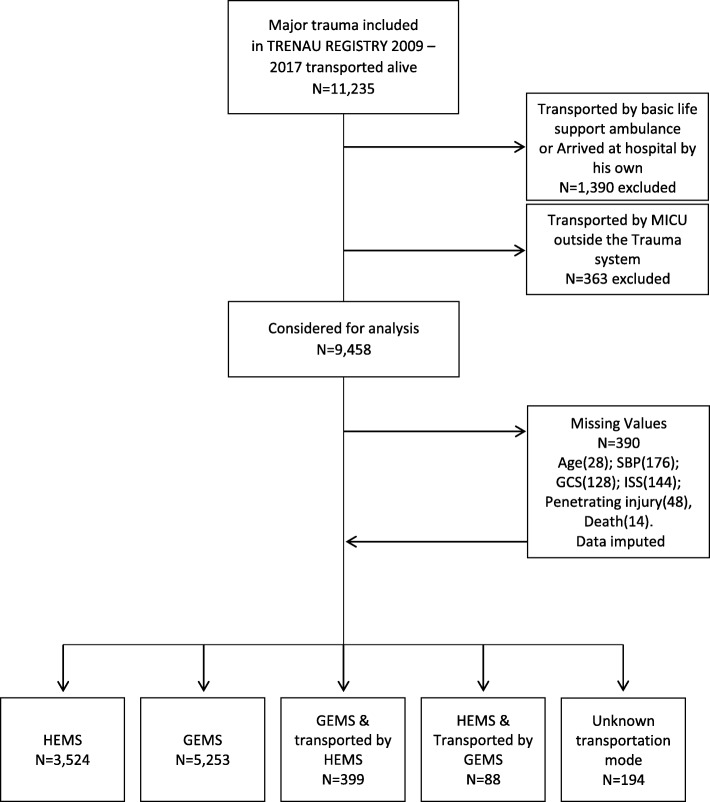
Flow chart of inclusion in the study

Overall, 9458 patients were included in this study (Fig. 1). There was 3524 (37%) helicopter patients and 5253 (56%) ground ambulance patients. Additionally, ground ambulance and helicopter were both dispatched for 487 (5%) patients for long transportation on request of ground ambulance already on-scene or due weather condition and difficulty of accessing patient by helicopter. Transportation was unknown for 194 (2%) patients who arrived alive at the trauma centre. Table 1 summarizes patient characteristics. The mean age was 38 years for all patients. Patients transported by ground ambulance had more penetrating injuries (9%) compared to helicopter patients (2%). helicopter treated more patients injured by falls (58%) and ground ambulance transported more patients injured by road traffic accident (61%). Mean ISS were similar in both groups. Intensity of prehospital resuscitation was not different between helicopter and ground ambulance (Table 1). Prehospital tracheal intubation was performed in 14% of patients in both groups. Median prehospital time from first call to arrival at hospital was longer in the helicopter group (95 min versus 85 min in the ground ambulance group, *P* < 0.001) (Table 2). The median on-scene medical time and the transport time were slightly longer in the helicopter group compared to the ground ambulance group. Sensitivity analysis did not show any discrepancies between complete case analysis and imputed missing values (Additional file 4). Injured patients initially cared by ground ambulance and secondarily transported by helicopter had more severe injuries compared to the ground ambulance -only group (Additional file 2). The minority of patients initially cared by helicopter but not transported by air, consisted of rare mountain injuries associated with unusual conditions (avalanche, high altitude alpinism, speleology, etc.). These cases were comparable among the helicopter and ground ambulance groups in terms of severity and intensity of the resuscitation (Additional file 2).

**Table 1:** Patient characteristics and prehospital therapy according to transportation mode

	Missing N (%)	All patients *N* = 9458	ground ambulance *N* = 5253	helicopter *N* = 3524	*P* Value
Mean age (years) (SD)	28 (0.3)	39 (20)	39 (20)	38 (19)	0.001
Sex male, N (%)	4 (0)	7281 (77)	3977 (76)	2768 (79)	0.002
Penetrating injury, N (%)	48 (0.5)	569 (6)	464 (9)	82 (2)	<0.001
Circumstances, N (%)	73 (0.8)				<0.001
Traffic accident		4109 (44)	3099 (59)	705 (20)	
Gunshots		148 (2)	100 (2)	38 (1)	
Stabbings		363 (4)	332 (6)	22 (1)	
Falls		3781 (40)	1382 (26)	2129 (61)	
Mountain sport accidents^a^, N (%)	72 (0.8)	2685 (28)	266 (5)	2253 (64)	<0.001
Injury severity score (ISS)	144 (1.5)				
Mean (SD)		16 (12)	16 (12)	16 (12)	<0.001
≥ 16, N (%)		4272 (46)	2243 (43)	1633 (47)	<0.001
Haemorrhagic shock, N (%)	175 (1.9)	375 (4)	218 (4)	116 (3)	0.116
Systolic blood pressure < 90 mmHg, N (%)	176 (1.9)	633 (7)	321 (6)	247 (7)	0.236
Glasgow coma scale ≤8, N (%)	128 (1.4)	1179 (13)	618 (12)	411 (12)	0.929
Severe traumatic brain injury, N (%)	131 (1.4)	1216 (13)	677 (13)	447 (13)	0.730
Prehospital procedure					
Intubation	48 (0.5)	1415 (15)	751 (14)	492 (14)	0.742
Fluid resuscitation > 1000 ml^b^	556 (5.9)	1158 (13)	644 (13)	394 (12)	0.251
Vasopressors	333 (3.5)	543 (6)	280 (5)	194 (6)	0.620
Chest tube or thoracostomy	334 (3.5)	106 (1)	60 (1)	35 (1)	0.548
Blood transfusion	335 (3.5)	129 (1)	61 (1)	50 (1)	0.259

SD: standard deviation. a Mountain sports included: Ski, Snowboard, Hiking, Mountain bike, Alpinism, Ice climbing, Climbing, Paragliding, Speed riding, Canyoning and Rafting.^b^ Crystalloids or colloids

Haemorrhagic shock was reported by the in-charge physician. Severe traumatic brain injury was defined by an head AIS ≥3

**Table 2:** Prehospital times according to transportation mode

	All patients Median [IQR] *N* = 9458	ground ambulance Median [IQR] BYE000730 *N* = 5253	helicopter Median [IQR] *N* = 3524	P Value
Response time	11 [7–19]	10 [6–16]	13 [8–21]	<0.001
Response medical time	26 [15–43]	23 [15–37]	30 [19–51]	<0.001
On scene medical time	34 [23–50]	32 [23–46]	35 [22–50]	0.001
Transport time	20 [14–31]	21 [14–32]	20 [13–30]	0.033
Total prehospital time	90 [67–120]	85 [63–113]	95 [72–124]	<0.001

BLS: Basic life Support (Fire department); IQR: inter quartile range

Response time (First call to arrival of the BLS ambulance)

Response medical time (First call to arrival of helicopter or ground ambulance)

On scene medical time (Arrival of helicopter or ground ambulance to departure from the scene)

Transport time (departure from the scene to arrival at hospital)

Total prehospital time (first call to arrival at hospital)

### Outcomes

**Fig. 2:**
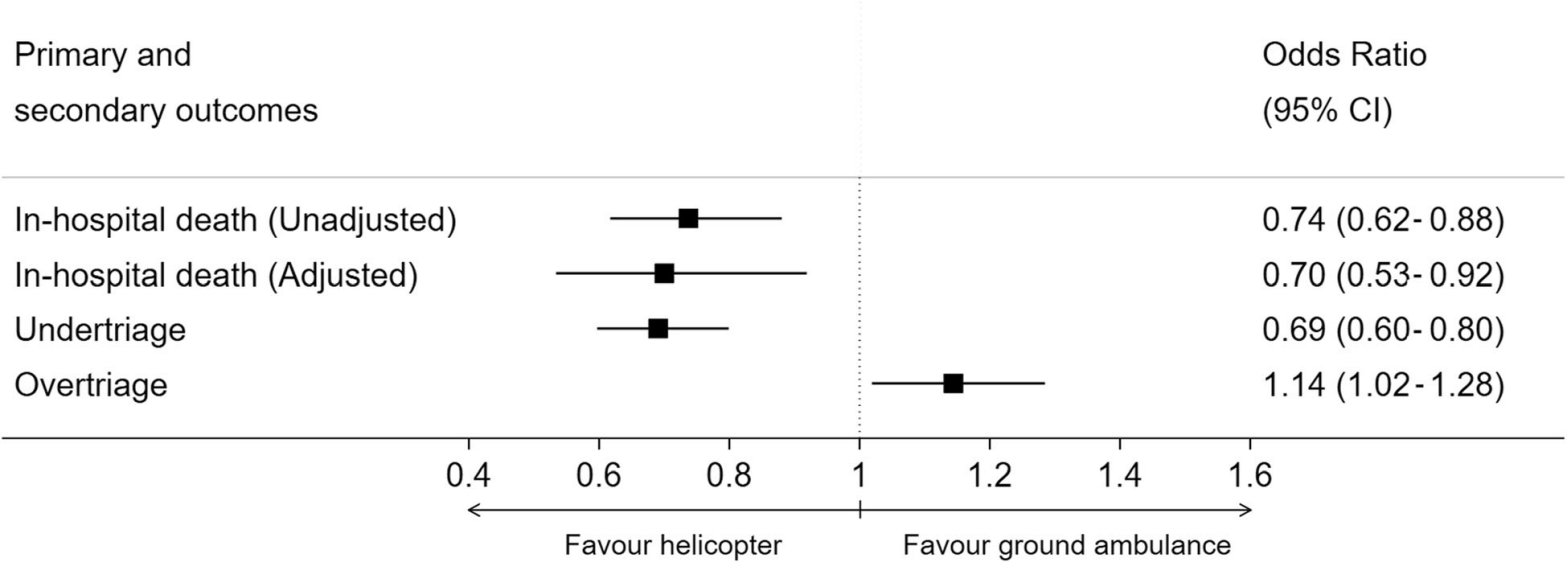
Primary and secondary outcomes

We observed 387 (7.4%) deaths in the ground ambulance group and 195 (5.5%) deaths in the helicopter group, corresponding to an absolute risk reduction (ARR) of − 1.8%; 95%CI (− 2.9; − 0.8); *P* < 0.001. The post-hoc power using this difference was 94%. After adjustment, the odds ratio (OR) of death in the helicopter group compared to the ground ambulance group was 0.70; 95% CI (0.53–0.92); *P* = 0.01 (Fig. 2). The entire model with all groups and predictors is presented in Additional file 3. The under-triage rate was lower in the helicopter group than ground ambulance group, respectively *n* = 377; 23%; 95%CI (21–25) vs *n* = 674; 30%; 95%CI (28–32); OR 0.69; 95%CI (0.60–0.80); *P* < 0.001. Over-triage was higher in the helicopter group than ground ambulance group, respectively *n* = 997; 53%; 95%CI (48–52) vs *n* = 872; 50%; 95%CI (48–52); OR 1.14; 95% CI (1.02–1.28); *P* = 0.02. Using the TRISS predictive score of mortality, the Standardized Mortality Ratio (SMR) was lower in the helicopter group than in the ground ambulance group (Table 3).

**Table 3:** Mortality assessed by TRISS and standardized mortality ratio

	ground ambulance *N* = 5253	helicopter *N* = 3524	P value
Number of death	387	195	
Observed mortality, %	7.4 (6.7–8.1)	5.5 (4.8–6.3)	
Expected mortality, %	8.3 (7.8–8.8)	8.2 (7.6–8.8)	
W	+ 1.1 (0.6–1.7)	+ 2.7 (2.1–3.2)	<0.001
Z	4.0	7.7	
M	0.87	0.87	
SMR	0.87 (0.80–0.93)	0.67 (0.60–0.74)	<0.001

SMR: standardized mortality ratio

Expected mortality calculated with the formula = 1 / (1 + exp.(−b)), where b = − 0.4499 + (0.8085 * RTS) + (− 0.0835 * ISS) + (− 1.7430 * age) / b = − 2.5355 + (0.9934 * rts) + (− 0.0651 * ISS) + (− 1.1360 * age). Age < 55 years = 0 and age ≥ 55 = 1

W is the difference between predicted number of survivors and the actual number of survivors, divided by the total number of cases divided by 100

Z score is the statistic compared with a standard normal distribution; null hypothesis is W = 0

M examine the similarity in the injury severities in the observed data compared to the predicted database

SMR = (number of observed death / number of expected death); Z-statistic, − 3.66

## Discussion

Our results show a reduction of mortality by one third in favour of helicopter compared to ground ambulance. Total prehospital time was longer with helicopter and the intensity of resuscitation was similar in both groups. Our results also demonstrated a reduction by one third of under-triage with helicopter compared to ground ambulance.

This study has important strengths. First, we used a prospective inception cohort including patients identified at an early prehospital stage. Second, we used rigorous methods to control for potential known confounders. We included continuous variables with their linear and polynomial terms in our model because an on-off step function for systolic blood pressure and age is biologically implausible. We performed logistic random effects regression to control for clustering. We also used a second prognostic model with the TRISS method that allowed us to estimate the standardised mortality ratio; results were similar with both methods, thereby improving confidence in the model. Third, our large sample size enabled calculation of precise effect estimates and a high post-hoc power of 94%.

There are limitations to our work that are worth noting. Dispatch of helicopter or ground ambulance was not randomly assigned, and despite adjustment with a multivariate model, unknown confounders might have influenced the findings. However, a randomized controlled trial would be difficult to conduct in this context as helicopter’s location depends of political and historical decision. Missing values represent another limitation to our study. We performed multiple imputation to not exclude any observation. Outcomes were missing for only 14 patients (0.001%) and predictors were missing in 390 patients (4%). Therefore, the overall proportion of missing values was low and complete case analysis would have been acceptable. However, as with many helicopter and ground ambulance studies, prehospital time is difficult to accurately capture in a trauma registry. We reported 20 to 50% of missing values in the different prehospital times. Multiple imputation could have led to bias if missing values are not at random. As we hypothesized that missing values in prehospital times would depend on the outcome, multiple imputation was considered more prudent than complete case analysis [20]. We report a sensitivity analysis in the Additional file; this analysis did not show any discrepancies. Finally, our results cannot be considered as generalizable. The Northern French Alps Emergency Network is an organised trauma system in a mountainous area. Winter ski resorts are usually distant from the regional trauma centre and ground transportation could take more than 2 h. For this reason, this area concentrates more helicopters than usual rural area. Injury patterns that occur in mountainous environments are also likely to differ from other populations served by helicopter worldwide.

Our results are consistent with findings from other recent studies. Galvagno et al. showed an increase of survival approximately 30% in a large study population using an American trauma registry using multiple statistical techniques to control for confounding including propensity score matching. They hypothesised that the benefit of helicopter transportation was not only due to speed but also attributed to highly trained medical crews [6]. A German trauma registry study also found a reduction of mortality of nearly 25% in favour of helicopter after adjustment of main confounders [21]. They hypothesised that rapid transportation enables rapid transport and access to care within the “golden hour.” In our study, we did not observe a difference in the intensity of initial prehospital resuscitation which is not a surprising finding since helicopter and ground ambulance crews have the same training and capabilities in our trauma system. We also observed that prehospital times were longer with helicopter compared to ground ambulance. Hence, we cannot explain the benefit observed by crew expertise or rapid transportation. Another important finding in our study is the reduction of undertriage with helicopter despite longer prehospital times. These results suggest that helicopter was used to transport patients to the regional trauma centre when ground transportation was too long to be considered. Helicopter allowed to increase the transportation distance and not to decrease the transportation time. In our organised trauma system, prehospital emergency physicians are well-versed and trained with triage protocols and are well-equipped to make accurate triage decisions [13]. However, long ground transportation remains an obstacle to direct admission at the regional trauma centre. From a public health perspective, a major goal of helicopter is to ensure equivalent outcomes for patients injured further from trauma centres compared to patients treated and transported by ground ambulance and injured in closer proximity to trauma centres. Miller et al. suggested that helicopters reduce rural and urban disparity by providing access to distant area and deployment of advanced medical team [22]. In our study, overtriage was slightly increased. This finding suggests that field triage was performed conservatively and is congruent with previous recommendations made by organizations such as the American College of Surgeons [15]. Hesselfeldt et al. showed that after the implementation of helicopter, time from the first call to arrival at the first hospital did not decrease but slightly increased [23]. Simultaneously, secondary inter hospital transfers decreased from 50 to 34%. Helicopter give to clinician opportunity to transport injured patient to the most appropriate facility. In doubt, clinicians usually choose transportation to regional trauma centre and contribute to increase overtriage.

Galvagno et al. suggested that the benefit of helicopter transportation is likely due to a combination of the crew’s expertise, rapid transportation over long distances and the fact that helicopter is part of an organized trauma system [10]. Our findings suggest that any benefit attributed to helicopter could be an accurate on-scene triage due to the possibility of a long-range transportation modality that allows for the application of appropriate field triage protocol.

Due to the nature of this study design, we cannot demonstrate a causal link. Further studies using causal mediation analysis might prove useful for confirming the reported mortality benefit associated with helicopter. As the helicopter is an expensive and limited resource, understanding the effectiveness of helicopter would help to improve the trauma system while avoiding unnecessary costs and safety risks. Additional formal cost-effectiveness studies would also help inform future refinements for field protocols authorizing helicopter transportation. Additional studies are recommended to identify specific dispatch criteria as the effect on reducing death appears to be offset when helicopter is secondarily dispatched [24].

## Conclusions

Helicopter transportation as part of an integrated trauma system was associated with a significant decrease in mortality for major trauma patients when helicopter was primarily dispatched. This observed benefit suggests that helicopter is the proper tool for long-distant transport allowing transportation to the regional trauma centre according to an established triage protocol in a rural area. Despite the limitations inherent with this work, policy makers should consider the result of this study in the context of rural and mountainous populations where time to definitive trauma care may be prolonged. Implementation of helicopter must be a thoughtful decision informed by an analysis the entire healthcare system including geographic designation of the trauma centre, transport times by transportation mode, triage rules and on-scene medical expertise.

## Supplementary information

**Additional file 1.** Trauma system of the Northern French Alps Emergency Network.

**Additional file 2.** Comparison between all groups of transportation.

**Additional file 3.** Logistic regression with in-hospital death as dependent variable and random effect on prehospital team.

**Additional file 4.** Prehospital times according to transportation mode including multiple imputation and complete case analysis as sensitivity analysis.

## Data Availability

The data that support the findings of this study are available from The Northern French Alps Emergency Network but restrictions apply to the availability of these data, which were used under license for the current study, and so are not publicly available. Data are however available from the authors upon reasonable request and with permission of The Northern French Alps Emergency Network.
