# Supplementary material for: Association of helicopter transportation and improved mortality for patients with major trauma in the northern French Alps trauma system: an observational study based on the TRENAU registry
Source: Scand J Trauma Resusc Emerg Med. 2020 May 12;28:35. doi: 10.1186/s13049-020-00730-z (PMC7218509; doi:10.1186/s13049-020-00730-z)
Supplement: Supplementary file 1 — Additional file 1. Trauma system of the Northern French Alps Emergency Network. [file 13049_2020_730_MOESM1_ESM.docx]

Additional file 1. Trauma system of the Northern French Alps Emergency Network

Switzerland

Italia

France

400 - 800

1200-2000

> 2000

800 - 1200

**Altitude (meter)**


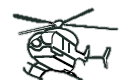


**Helicopter**


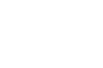


**ALS ground ambulance**

**Regional Trauma Center**


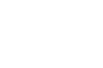

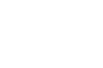

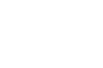

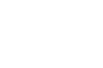

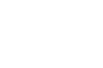

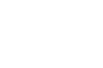

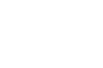

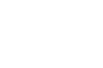

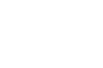

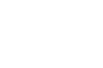

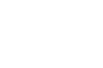

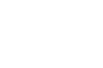

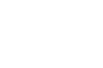


**Grenoble**

**Chambéry**

**Annecy**


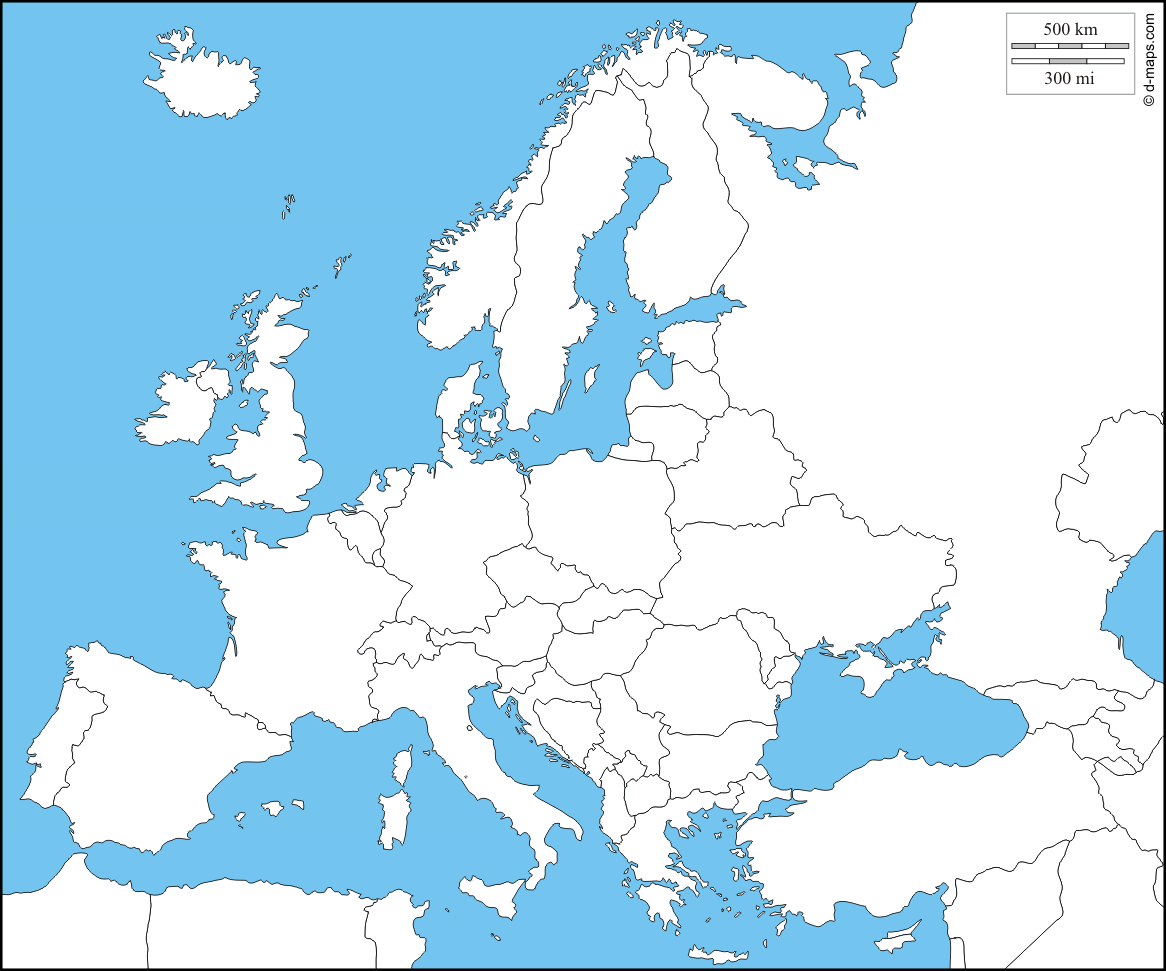

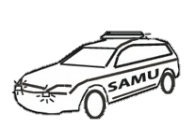

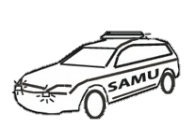

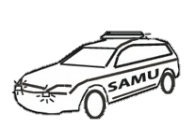

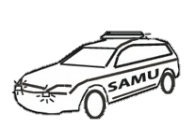

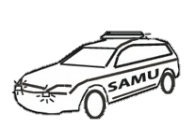

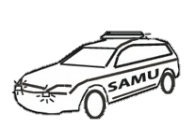

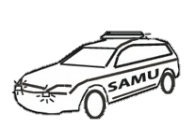

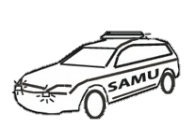

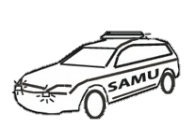

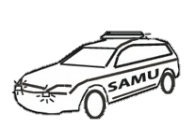

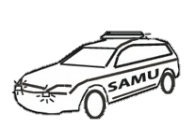

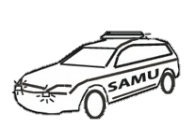

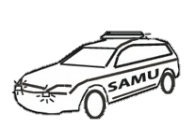

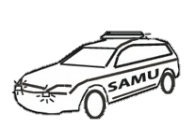

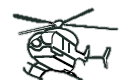

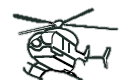

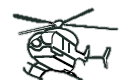

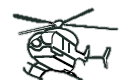

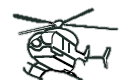

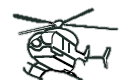

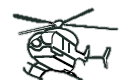


**Mobile Intensive Care Unit**
