# Supplementary material for: Association of helicopter transportation and improved mortality for patients with major trauma in the northern French Alps trauma system: an observational study based on the TRENAU registry
Source: Scand J Trauma Resusc Emerg Med. 2020 May 12;28:35. doi: 10.1186/s13049-020-00730-z (PMC7218509; doi:10.1186/s13049-020-00730-z)
Supplement: Supplementary file 2 — Additional file 2. Comparison between all groups of transportation. [file 13049_2020_730_MOESM2_ESM.docx]

Additional file 2. Comparison between all groups of transportation.

|  | All patients  N= 9,458 | Ground ambulance  N= 5,253 | Helicopter  N= 3,524 | Ground then helicopter  N=399 | Helicopter then ground  N=88 |
| --- | --- | --- | --- | --- | --- |
| Mean age (years) (SD) | 39 (20) | 39 (20) | 38 (19) | 41(20) | 40 (19) |
| Sex male, N (%) | 7,281 (77) | 3,977 (76) | 2,768 (79) | 324 (81) | 64 (73) |
| Mechanism, N (%) |  |  |  |  |  |
| Penetrating | 569 (6) | 464 (9) | 82 (2) | 15 (4) | 1 (11) |
| Circumstances, N (%) |  |  |  |  |  |
| Traffic accident | 4,109 (44) | 3,099 (59) | 705 (20) | 223 (56) | 20 (23) |
| Gunshots | 148 (2) | 100 (2) | 38 (1) | 8 (2) | 1 (1) |
| Stabbings | 363 (4) | 332 (6) | 22 (1) | 3 (1) | 0 |
| Falls | 3,781 (40) | 1,382 (26) | 2,129 (61) | 132 (33) | 49 (56) |
| Mountain sport accidents, N (%) | 2,685 (28) | 266 (5) | 2,253 (64) | 53 (13) | 54 (61) |
| Injury severity score (ISS) |  |  |  |  |  |
| Mean (sd) | 16 (12) | 16 (12) | 16 (12) | 21 (13) | 14 (11) |
| ≥ 16, N (%) | 4,272 (46) | 2,243 (43) | 1,633 (47) | 256 (64) | 36 (42) |
| Haemorrhagic shock, N (%) | 375 (4) | 218 (4) | 116 (3) | 30 (8) | 4 (5) |
| Systolic blood pressure < 90 mmHg, N (%) | 633 (7) | 321 (6) | 247 (7) | 43 (11) | 5 (6) |
| Glasgow coma scale ≤ 8, N(%) | 1,179 (13) | 618 (12) | 411 (12) | 102 (25) | 9 (10) |
| Severe traumatic brain injury, N (%) | 1,216 (13) | 677 (13) | 447 (13) | 66 (17) | 7 (8) |
| Prehospital Tracheal intubation, N (%) | 1,415 (15) | 751 (14) | 492 (14) | 129 (32) | 12 (14) |
| Median total prehospital time [IQR] | 90 [67-120] | 85 [63-113] | 95 [72-124] | 110 [87-136] | 105 [76-152] |
| In-hospital death, N (%) | 660 (7.0) | 385 (7.4) | 195 (5.5) | 52 (13.0) | 8 (9.1) |
| Undertriage, N (%) | 1,138 (26) | 683 (30) | 377 (23) | 34 (13) | 15 (41) |
| Overtriage, N (%) | 2,664 (52) | 1,485 (50) | 997 (53) | 107 (76) | 26 (51) |

SD: standard deviation. Mountain sports included Ski, Snowboard, Hiking, Mountain bike, Alpinism, Ice climbing, Climbing, Paragliding, Speed riding, Canyoning and Rafting.

Haemorrhagic shock was reported by the in-charge physician. Severe traumatic brain injury was defined by an head AIS ≥3.
