# Supplementary material for: Association of helicopter transportation and improved mortality for patients with major trauma in the northern French Alps trauma system: an observational study based on the TRENAU registry
Source: Scand J Trauma Resusc Emerg Med. 2020 May 12;28:35. doi: 10.1186/s13049-020-00730-z (PMC7218509; doi:10.1186/s13049-020-00730-z)
Supplement: Supplementary file 3 — Additional file 3. Logistic regression with in-hospital death as dependent variable and random effect on prehospital team. [file 13049_2020_730_MOESM3_ESM.docx]

Additional file 3. Logistic regression with in-hospital death as dependent variable and random effect on prehospital team.

|  | OR | 95% CI | P value |
| --- | --- | --- | --- |
| Transportation mode |  |  |  |
| Ground ambulance | 1 |  |  |
| Helicopter | 0.70 | 0.53 – 0.92 | 0.009 |
| Ground ambulance then transport by helicopter | 1.06 | 0.67-1.67 | 0.800 |
| Helicopter then transport by ground ambulance | 2.49 | 0.88-7.01 | 0.085 |
| SBP | 0.95 | 0.94-0.97 | <0.001 |
| SBP^2^ | 1.00 | 1.00-1.00 | <0.001 |
| ISS | 1.32 | 1.22-1.43 | <0.001 |
| ISS^2^ | 0.99 | 0.99-0.99 | <0.001 |
| ISS^3^ | 1.00 | 1.00-1.00 | <0.001 |
| GCS | 0.22 | 0.12-0.41 | <0.001 |
| GCS^2^ | 1.14 | 1.05-1.23 | 0.002 |
| GCS^3^ | 0.99 | 0.99-0.99 | 0.013 |
| Age | 0.94 | 0.92-0.97 | <0.001 |
| Age^2^ | 1.00 | 1.00-1.00 | <0.001 |
| Sex male | 1.34 | 1.05-1.78 | 0.026 |
| Total prehospital time | 1.00 | 0.99-1.00 | 0.903 |
| Circumstances |  |  |  |
| Road traffic accident | 1 |  |  |
| Penetrating injury | 4.34 | 2.57-7.34 | <0.001 |
| Fall | 1.27 | 0.98-1.64 | 0.074 |

OR: Odds ratio; CI: confidence interval; HEMS: Helicopter emergency medical service; GEMS: Ground emergency medical service; SBP: Initial systolic blood pressure; GCS: initial Glasgow coma scale.

N total = 9,458; GEMS (N=5,253); HEMS (N=3,524); GEMS first and transported by HEMS (N=399); HEMS first and transported by GEMS (N=88); Unknown (N=194).

R^2^=0.34; C-statistic 0.96, 95%CI (0.95-0.97), overall calibration 1.00 95%CI(0.95-1.05).

SBP^2^, ISS^2^ GCS^2^, Age^2^ represent the quadratic term; ISS^3^, GCS^3^ represent the cubic term.
