# Supplementary material for: Association of helicopter transportation and improved mortality for patients with major trauma in the northern French Alps trauma system: an observational study based on the TRENAU registry
Source: Scand J Trauma Resusc Emerg Med. 2020 May 12;28:35. doi: 10.1186/s13049-020-00730-z (PMC7218509; doi:10.1186/s13049-020-00730-z)
Supplement: Supplementary file 4 — Additional file 4. Prehospital times according to transportation mode including multiple imputation and complete case analysis as sensitivity analysis. [file 13049_2020_730_MOESM4_ESM.docx]

Additional file 4. Prehospital times according to transportation mode including multiple imputation and complete case analysis as sensitivity analysis.

|  | All patients  Median [IQR] | Ground ambulance  Median [IQR] | Helicopter  Median [IQR] | P Value |
| --- | --- | --- | --- | --- |
| Response time |  |  |  |  |
| Complete case analysis (N=4,074) | 11 [7-17] | 10 [6-15] | 15 [8-18] | <0.001 |
| Multiple imputation (N=9,458) | 11 [7-19] | 10 [6-16] | 13 [8-21] | <0.001 |
| Response medical time |  |  |  |  |
| Complete case analysis (N=7,080) | 25 [15-40] | 22 [15-34] | 30 [20-50] | <0.001 |
| Multiple imputation (N=9,458) | 26 [15-43] | 23 [15-37] | 30 [19-51] | <0.001 |
| On scene medical time |  |  |  |  |
| Complete case analysis (N=6,387) | 34 [25-49] | 33 [25-45] | 35 [24-50] | <0.001 |
| Multiple imputation (N=9,458) | 34 [23-50] | 32 [23-46] | 35 [22-50] | 0.001 |
| Transport time |  |  |  |  |
| Complete case analysis (N=6,498) | 20 [14-31] | 22 [15-33] | 20 [13-30] | <0.001 |
| Multiple imputation (N=9,458) | 20 [14-31] | 21 [14-32] | 20 [13-30] | 0.033 |
| Total prehospital time |  |  |  |  |
| Complete case analysis (N=7,355) | 90 [67-117] | 83 [62-110] | 95 [73-122] | <0.001 |
| Multiple imputation (N=9,458) | 90 [67-120] | 85 [63-113] | 95 [72-124] | <0.001 |

IQR: inter quartile range.

Response time (First call to arrival of the first ambulance)

Response medical time (First call to arrival of helicopter or ground ambulance)

On scene medical time (Arrival of helicopter or ground ambulance to departure from the scene)

Transport time (departure from the scene to arrival at hospital)

Total prehospital time (first call to arrival at hospital)
